# Prevalence of and Inequities in Poor Mental Health Across 3 US Surveys, 2011 to 2022

**DOI:** 10.1001/jamanetworkopen.2024.54718

**Published:** 2025-01-15

**Authors:** Emily Wright, Emily C. Dore, Karestan C. Koenen, Christina Mangurian, David R. Williams, Rita Hamad

**Affiliations:** 1 Department of Social and Behavioral Sciences, Harvard T.H. Chan School of Public Health, Boston, Massachusetts; 2 Department of Epidemiology, Harvard T.H. Chan School of Public Health, Boston, Massachusetts; 3 Department of Psychiatry and Behavioral Sciences, UCSF Weill Institute for Neurosciences, University of California, San Francisco

## Abstract

This survey study uses 3 nationally representative US surveys to evaluate the prevalence of poor mental health overall and by age, sex, and race and ethnicity.

## Introduction

Prior literature suggests mental health issues in the United States have not only increased population-wide in recent decades but also accumulated unevenly and unjustly across social groups.^1,2,3^ However, few studies have leveraged multiple national surveys to strengthen understanding of the changing burden of poor mental health and differences across social groups. Such analyses may yield useful insights for clinical care, public health, and policies and interventions to address new, dynamic poor mental health patterns. To address this gap, we quantified the prevalence of poor mental health—and inequities by age, sex, and racial and ethnic group—among US adults across 3 national health surveys.

## Methods

In this survey study, we analyzed 2011 to 2022 data from 3 nationally representative repeated cross-sectional surveys: the Behavioral Risk Factor Surveillance System (BRFSS), National Survey on Drug Use and Health (NSDUH), and National Health Interview Survey (NHIS). See key survey features in eTables 1 and 2 in Supplement 1.

In NSDUH and NHIS, we examined past 30-day moderate-to-serious psychological distress by dichotomizing Kessler-6 scores as 0 to 4 vs 5 to 24 (higher scores indicate greater distress).^4^ In BRFSS, we assessed whether respondents reported 1 or more poor mental health days in the past 30 days because BRFSS does not include the Kessler-6.^5^ We refer to both outcomes as metrics of poor mental health.

After examining the weighted characteristics of adults in each survey (eTable 3 in Supplement 1), we estimated poor mental health prevalence by survey year overall and by age, sex, and self-reported racial and ethnic group. All analyses accounted for complex survey designs. We did not test for time trends because of discontinuities in NSDUH (pandemic-related) and NHIS (survey redesign–related) data comparability over time, even after weighting (eTable 2 in Supplement 1). Since less than 3% of observations were missing for each variable, we conducted complete case analysis. This secondary analysis of public-use, deidentified survey data was considered not human subjects research by the Harvard University Committee on the Use of Human Subjects. We followed the AAPOR reporting guideline. Stata version 18.0 (StataCorp) was used for analysis. The eAppendix in Supplement 1 provides additional methodological details.

## Results

**Figure 1.**
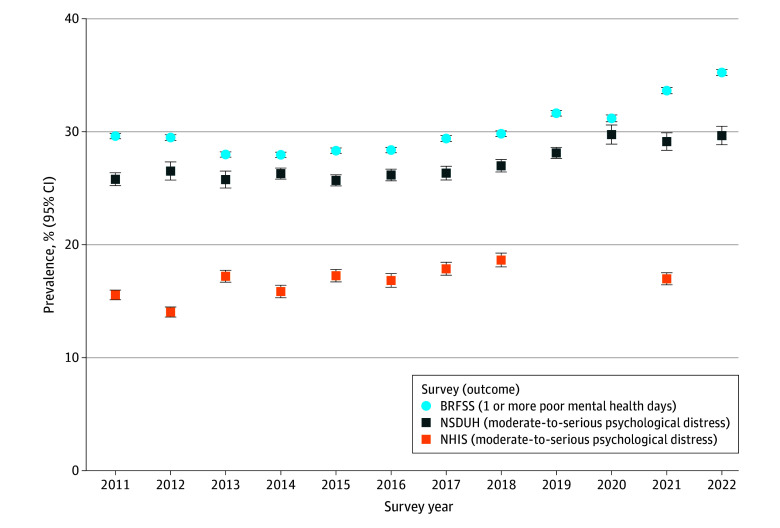
Weighted Prevalence of Poor Mental Health by Survey: US Adults, 2011 to 2022 Data on psychological distress were not collected by the National Health Interview Survey (NHIS) in 2019, 2020, and 2022 due to survey redesign. Whiskers indicate 95% CIs. BRFSS indicates Behavioral Risk Factor Surveillance System; NSDUH, National Survey on Drug Use and Health.

Weighted characteristics of the US adult population for 2011 to 2022 were similar in the 3 surveys: approximately 3 billion adults, with approximately 13% aged 18 to 25 years, 51% female, 16% Hispanic, 5% non-Hispanic Asian, 12% non-Hispanic Black, and 64% non-Hispanic White. From 2011 to 2022, 35.7% to 42.5% of adults in BRFSS experienced poor mental health. Similarly, 31.1% to 35.8% experienced poor mental health in NSDUH, but only 18.7% to 20.5% did in NHIS, despite these latter 2 surveys using the same psychological distress measure (Figure 1). Given differences in BRFSS and NSDUH vs NHIS (and NHIS’s inconsistent mental health data collection in recent years) the remaining results focus on BRFSS and NSDUH.

**Figure 2.**
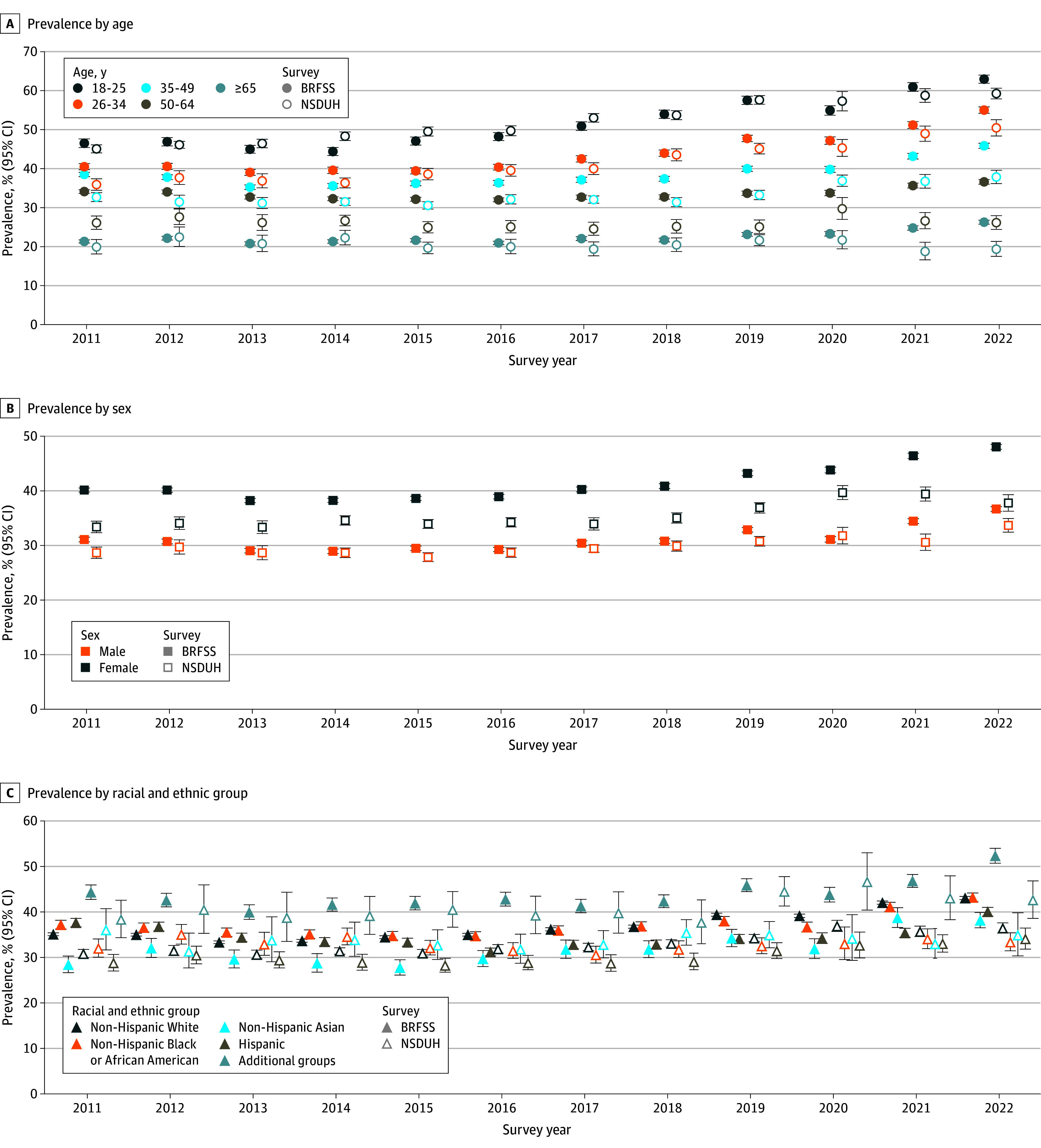
Weighted Prevalence of Poor Mental Health by Social Group: US Adults, 2011-2022 The poor mental health outcomes assessed are 1 or more poor mental health days in the Behavioral Risk Factor Surveillance System (BRFSS) and moderate-to-serious psychological distress in the National Survey on Drug Use and Health (NSDUH). Due to limits on public-use data harmonization, in BRFSS, the first 2 age categories include, respectively, those aged 18 to 24 years and those aged 25 to 34 years. The category additional racial and ethnic groups refers to groups collapsed due to small size. In BRFSS, this includes American Indian or Alaskan Native only, non-Hispanic; Native Hawaiian or other Pacific Islander only, non-Hispanic; other race only, non-Hispanic; and multiracial, non-Hispanic. In NSDUH, this includes American Indian or Alaska Native only, non-Hispanic; Native Hawaiian or Other Pacific Islander only, non-Hispanic; and more than 1 race, non-Hispanic. Overlapping estimates were horizontally dodged for improved visibility. Whiskers indicate 95% CIs.

In subgroup analyses, poor mental health prevalence was higher among younger adults and lower among older adults—and increasingly so, until 2020, when younger adult prevalence stabilized or decreased in NSDUH and BRFSS, respectively, while older adult prevalence increased or remained stable (Figure 2A). Poor mental health prevalence was consistently higher among female vs male adults. This gap widened in 2020 to 2021, then decreased in 2022 in both surveys (Figure 2B). Some racial and ethnic inequities narrowed or reversed direction, driven by worsening mental health among White adults (Figure 2C). For example, prevalence was higher among Black adults than White adults in both surveys from 2011 to 2015, but this gap reversed direction thereafter, most notably in 2020.

## Discussion

This survey study documents increasingly prevalent poor mental health from 2011 to 2022 across multiple US health surveys, with notable prevalence differences in BRFSS and NSDUH vs NHIS.^6^ Inequities in these outcomes by age, sex, and racial and ethnic group were often sizeable and changed over time in distinct ways, consistent with findings in prior literature.^1,2,3^ Study limitations included our use of similar, but not directly comparable, measures of nonspecific poor mental health across surveys. Further research on mechanisms underlying observed trends before and since 2020 is needed to inform policy and clinical interventions to improve population mental health and health equity.

## Notes

Supplement 1. **eAppendix.** Supplemental Methods **eTable 1.** Selected Features of Each Survey Before the Onset of the COVID-19 Pandemic **eTable 2.** Selected Impacts of the COVID-19 Pandemic on Each Survey **eTable 3.** Weighted Sample Characteristics by Survey: US Adults, 2011-2022
eReferences.

Supplement 2.
Data Sharing Statement
