# Supplementary material for: Prevalence of and Inequities in Poor Mental Health Across 3 US Surveys, 2011 to 2022
Source: JAMA Netw Open. 2025 Jan 15;8(1):e2454718. doi: 10.1001/jamanetworkopen.2024.54718 (PMC11736504; doi:10.1001/jamanetworkopen.2024.54718)
Supplement: Supplement 1. — eAppendix. Supplemental Methods eTable 1. Selected Features of Each Survey Before the Onset of the COVID-19 Pandemic eTable 2. Selected Impacts of the COVID-19 Pandemic on Each Survey eTable 3. Weighted Sample Characteristics by Survey: US Adults, 2011-2022 eReferences. [file jamanetwopen-e2454718-s001.pdf]

Supplemental Online Content

Wright E, Dore EC, Koenen KC, Mangurian C, Williams DR, Hamad R. Prevalence of and inequities in poor mental health across 3 US surveys, 2011 to 2022. *JAMA Netw Open.* 2025;8(1):e2454718. doi:10.1001/jamanetworkopen.2024.54718

eAppendix. Supplemental Methods

eTable 1. Selected Features of Each Survey Before the Onset of the COVID-19 Pandemic

eTable 2. Selected Impacts of the COVID-19 Pandemic on Each Survey

eTable 3. Weighted Sample Characteristics by Survey: US Adults, 2011-2022

eReferences.

This supplemental material has been provided by the authors to give readers additional information about their work.

© 2024 Wright E et al. *JAMA Network Open*.

eAppendix. Supplemental Methods

Social groups

Sex

Data on sex were interviewer-assessed in the National Survey on Drug Use and Health (NSDUH) from 2011-2019, a combination of interviewer-assessed and respondent-reported in 2020-2021, and fully respondent-reported in 2022. In the National Health Interview Survey (NHIS), sex was interviewer-assessed from 2011-2012 and respondent-reported from 2013-2022. In the Behavioral Risk Factor Surveillance System (BRFSS), sex was interviewer-assessed from 2011-2015 and respondent-reported from 2016-2022.

Racialized group

Data on racialized group were collected using different questions with different survey-provided response options across the three surveys and over time. The categories for "White, non-Hispanic (NH)", "Black or African American, NH", and "Asian, NH" include individuals who identified that race only (i.e., not multiracial individuals). Our analytic category of "Additional racialized groups collapsed due to small size" category includes, in BRFSS, "American Indian or Alaskan Native only, NH", "Native Hawaiian or other Pacific Islander only, NH", "Other race only, NH", and "Multiracial, NH." In NSDUH, this category includes "American Indian or Alaska Native only, NH", "Native Hawaiian or Other Pacific Islander only, NH", and "More than one race, NH". In NHIS, it includes "American Indian/Alaska Native only, NH", "Other race", "Multiple race", and "Race group not releasable."

Statistical analyses

All analyses of prevalence were conducted in Stata (Version 18.0) using *svyset* and *svy* commands to account for the complex design of each survey (i.e., using the weighting and variance estimation variables provided by each survey).[1] We used logit-transformed 95% confidence intervals (CI), which are constrained to the parameter space of a prevalence. For NSDUH and NHIS, we used the *subpop* option to estimate prevalences among sampled adults without losing the information about sampled children needed to estimate standard errors. We plotted prevalences and their 95% CI using R (Version 4.3.1) and the *haven*, *ggplot2*, *dplyr*, and *cowplot* packages.[2–6] Overlapping estimates were horizontally dodged for improved visibility. We followed AAPOR reporting guidelines for survey studies by providing information on key BRFSS, NSDUH, and NHIS methods and results (e.g., population under study, questionnaire wording, modes of data collection, weighting procedures).[7]

eTable 1. Selected Features of Each Survey Before the Onset of the COVID-19 Pandemic

Topic	NSDUH	NHIS	BRFSS
**Population**[a]	Civilian, non-institutionalized population aged 12 or older of the United States<br>- Includes residents of households and non-institutional group quarters, as well as civilians on military bases	Civilian, non-institutionalized population (all ages) of the United States[8]<br>- Includes residents of households and non-institutional group quarters<br>- Excludes civilians living on military bases	Non-institutionalized adult population (≥18 years) of the United States[9] living in households or college housing with landline telephones or a working cellular telephone<br>- No mention of civilian status
**Geographies included**	All 50 states and Washington, DC	All 50 states and Washington, DC[8]	All 50 states, Washington, DC, Guam, Puerto Rico, and the U.S. Virgin Islands[9]
**Languages in which survey is conducted**	English, Spanish	English, Spanish	English, Spanish (and additional languages [e.g., Mandarin, Portuguese] in some states[10,11])
**Survey modality for mental health questions**	Audio computer-assisted self-interviewing[12]	Computer-assisted face-to-face interviewing[13]	Computer-assisted telephone interviewing
**Order and phrasing of questions for selected mental health outcomes**	During the past 30 days, how often did you feel…<br>1) nervous?<br>2) hopeless?<br>3) restless or fidgety?<br>4) so sad or depressed that nothing could cheer you up?<br>5) that everything was an effort?<br>6) down on yourself, no good or worthless?[14]	During the past 30 days, how often did you feel…<br>1) so sad that nothing could cheer you up?<br>2) nervous?<br>3) restless or fidgety?<br>4) hopeless?<br>5) that everything was an effort?<br>6) worthless?[15]	Now thinking about your mental health, which includes stress, depression, and problems with emotions, for how many days during the past 30 days was your mental health not good?[16]

Abbreviations: BRFSS = Behavioral Risk Factor Surveillance System; NHIS = National Health Interview Survey; NSDUH = National Survey on Drug Use and Health.
[a] Only sampled adults are included in the present analysis.

eTable 2. Selected Impacts of the COVID-19 Pandemic on Each Survey

Topic	NSDUH	NHIS[b]	BRFSS
**Interruption of data collection**	Yes<br>- No data collected from March-September 2020[17]	No[18]	Yes<br>- 16 states began data collection late (between February and July)[19]<br>- 23 states did not collect a full set of 12 monthly landline and/or cellphone samples[19]<br>- In 2021, Florida was unable to collect enough data to meet minimum requirements for inclusion in 2021 BRFSS public-use data set[20]
**Survey modality change**	Yes	Yes	No
Pre-pandemic modality	In-person (with computer-assisted self-interviewing for sensitive questions)	In-person	Telephone (landline and cellphone)
Post-pandemic-onset 2020 modalities	Web (93.0%) + in-person (7.0%)[21]	Telephone (monthly: 70-100%)[18] + in-person (0-30%)	
2021 modalities	Web (54.6%) + in-person (45.4%)[22]	Telephone (62.8%)[23] + in-person (37.2%)	
2022 modalities	Web (42.4%) + in-person (57.6%)[22]	Telephone (55.7%)[24] + in-person (44.3%)	
**Breaks in survey comparability**	Yes<br>- 2020 data incomparable with pre-2020 data<br>- 2021 data incomparable with prior data[22,25]	No[18]	No[19]
**2019 response rate[a]**	46.90%[17]	59.1%[8]	Median state response rate: 49.4%[26]
**2020 response rate[a]**	9.84%[27]	48.9%[18]	Median: 47.9%[28]
**2021 response rate[a]**	9.91%	50.9%[23]	Median: 44.0%[20]
**2022 response rate[a]**	11.91%	47.7%[24]	Median: 45.1%[29]
**Additional notes**		Approximately half of the original sample allocated for the last five months of 2020 was replaced with adults who had completed the 2019 NHIS Sample Adult interview (i.e., longitudinal data collection)[18]	

Abbreviations: BRFSS = Behavioral Risk Factor Surveillance System; NHIS = National Health Interview Survey; NSDUH = National Survey on Drug Use and Health.
[a] Response rates provided for the NSDUH are the unweighted Overall Response Rates. Response rates provided for NHIS are the unweighted Final Adult Sample Response Rate. Response rates provided for BRFSS are the unweighted Combined Landline Telephone and Cellular Telephone Response Rate. Because BRFSS is a state-based survey, we provide the median survey response rate was for all states, territories, and Washington, DC. [b] Due to methodological changes involved in the 2019 NHIS redesign, data on multiple health outcomes (including mental health) pre- versus post-2019 cannot be compared.

© 2024 Wright E et al. *JAMA Network Open*.

eTable 3. Weighted Sample Characteristics by Survey: US Adults, 2011-2022			
Characteristic	BRFSS 2011-2022 N=3,018,470,811[a]	NSDUH 2011-2022 N=2,941,200,993[a]	NHIS 2011-2022 N=2,938,619,280[a]
**Age (years)**			
18-25[b]	12.7	14.0	13.8
26-34[b]	17.6	15.9	16.0
35-49	23.9	25.0	24.9
50-64	25.5	25.2	25.4
≥65	20.4	19.9	19.9
**Sex**			
Male	48.7	48.3	48.3
Female	51.3	51.7	51.7
**Racialized group**			
White, NH	62.8	64.1	64.4
Black or African American, NH	11.7	11.8	11.7
Asian, NH	5.2	5.5	5.7
Hispanic	17.0	15.9	15.9
Additional racialized groups collapsed due to small size	3.2	2.6	2.4
**Education[c]**			
Bachelor's degree or more	27.5	31.6	31.1
Some college or Associate's degree	30.7	30.6	30.4
High school diploma	28.2	25.8	26.3
Less than high school diploma	13.7	11.9	12.3
**Number of poor mental health days, past 30 days**			
0	63.5	Not collected[e]	Not collected
1-30	36.5	Not collected	Not collected
**Moderate-to-serious psychological distress, past 30 days**	Not collected[d]	32.8	20.1

Abbreviations: BRFSS = Behavioral Risk Factor Surveillance System; NH = Non-Hispanic; NHIS = National Health Interview Survey; NSDUH = National Survey on Drug Use and Health.

[a] Weighted (i.e., representative of the US adult population) number of individuals in all study years (2011-2022). The unweighted number of individuals was 5,457,853 for BRFSS, 492,163 for NSDUH, and 378,350 for NHIS.

[b] The most granular public-use age variables in NSDUH and BRFSS cannot be fully harmonized. As a result, the first two age categories for BRFSS are as follows: 18-24 and 25-34 years.

[c] Prior to 2015, NSDUH asked a different question about the number of grades/years of school completed.[68] Percents for NSDUH on this variable are, thus, regarding 2015-2022 data.

[d] Data on psychological distress were not collected in BRFSS during the study years.

[e] Data on poor mental health days were not collected in either NSDUH or NHIS.

© 2024 Wright E et al. *JAMA Network Open*.

eReferences

1. StataCorp. Stata Statistical Software: Release 18. Published online 2023.
2. R Core Team. R: A Language and Environment for Statistical Computing. Published online 2023. https://www.R-project.org/
3. Wickham H, Miller E, Smith D. haven: Import and Export “SPSS”, “Stata” and “SAS” Files. Published online 2023. https://CRAN.R-project.org/package=haven
4. Wickham H. ggplot2: Elegant Graphics for Data Analysis. Published online 2016.
5. Claus WO. cowplot: Streamlined Plot Theme and Plot Annotations for “ggplot2.” Published online 2024. https://CRAN.R-project.org/package=cowplot
6. Wickham H, François R, Henry L, Müller K, Vaughan D. dplyr: A Grammar of Data Manipulation. Published online 2023. https://CRAN.R-project.org/package=dplyr
7. American Association for Public Opinion Research. Best Practices for Survey Research - AAPOR. Accessed October 17, 2024. https://aapor.org/standards-and-ethics/best-practices/
8. National Center for Health Statistics. Survey Description, National Health Interview Survey, 2019. Hyattsville, MD. 2020.
9. Centers for Disease Control and Prevention. Overview, Behavioral Risk Factor Surveillance System, 2019. Atlanta, GA: U.S. Department of Health and Human Services, Centers for Disease Control and Prevention; 2019.
10. Massachusetts Department of Public Health. Behavioral Risk Factor Surveillance System (BRFSS) Data. Population Health Information Tool. Accessed February 29, 2024. https://www.mass.gov/info-details/behavioral-risk-factor-surveillance-system-brfss-data
11. Pickens CM. Surveillance for certain health behaviors and conditions among states and selected local areas — Behavioral Risk Factor Surveillance System, United States, 2015. *MMWR Surveill Summ*. 2018;67(9):1-90. doi:10.15585/mmwr.ss6709a1
12. Center for Behavioral Health Statistics and Quality. *2019 National Survey on Drug Use and Health (NSDUH): CAI Specifications for Programming (English Version)*. Rockville, MD: Substance Abuse and Mental Health Services Administration; 2018.
13. National Center for Health Statistics. Survey Description, National Health Interview Survey, 2018. Hyattsville, MD. 2019.
14. Center for Behavioral Health Statistics and Quality. *2019 National Survey on Drug Use and Health: Methodological Summary and Definitions*. Rockville, MD: Substance Abuse and Mental Health Services Administration; 2020.
15. National Center for Health Statistics. *2018 NHIS Questionnaire - Sample Adult*. 2019. Accessed February 29, 2024. https://ftp.cdc.gov/pub/Health_Statistics/NCHS/Survey_Questionnaires/NHIS/2018/english/qadult.pdf

© 2024 Wright E et al. *JAMA Network Open*.

16. Centers for Disease Control and Prevention. *2018 BRFSS English Questionnaire*. 2018. Accessed March 19, 2024. https://www.cdc.gov/brfss/questionnaires/pdf-ques/2018_BRFSS_English_Questionnaire.pdf

17. Center for Behavioral Health Statistics and Quality. *2020 National Survey on Drug Use and Health (NSDUH) Methodological Resource Book, Section 8: Data Collection Final Report*. Substance Abuse and Mental Health Services Administration; 2022.

18. National Center for Health Statistics. Survey Description, National Health Interview Survey, 2020. Hyattsville, MD. 2021.

19. Centers for Disease Control and Prevention. *Comparability of Data BRFSS 2020*. 2021. Accessed February 27, 2024. https://www.cdc.gov/brfss/annual_data/2020/pdf/compare-2020-508.pdf

20. Centers for Disease Control and Prevention. *Behavioral Risk Factor Surveillance System: 2021 Summary Data Quality Report*. 2022. Accessed February 27, 2024. https://www.cdc.gov/brfss/annual_data/2021/pdf/2021-dqr-508.pdf

21. Center for Behavioral Health Statistics and Quality. *2020 National Survey on Drug Use and Health (NSDUH): Methodological Summary and Definitions*. Substance Abuse and Mental Health Services Administration; 2021.

22. Center for Behavioral Health Statistics and Quality. *2022 National Survey on Drug Use and Health (NSDUH): Methodological Summary and Definitions*. Substance Abuse and Mental Health Services Administration; 2023.

23. National Center for Health Statistics. Survey Description, National Health Interview Survey, 2021. Hyattsville, MD. 2022.

24. National Center for Health Statistics. Survey Description, National Health Interview Survey, 2022. Hyattsville, MD. 2023.

25. Substance Abuse and Mental Health Services Administration. National Survey on Drug Use and Health 2020. Data Sources. Accessed February 29, 2024. https://www.datafiles.samhsa.gov/dataset/national-survey-drug-use-and-health-2020-nsduh-2020-ds0001

26. Centers for Disease Control and Prevention. *Behavioral Risk Factor Surveillance System: 2019 Summary Data Quality Report*. 2020. Accessed February 27, 2024. https://www.cdc.gov/brfss/annual_data/2019/pdf/2019-sdqr-508.pdf

27. Center for Behavioral Health Statistics and Quality. *2022 National Survey on Drug Use and Health (NSDUH) Methodological Resource Book, Section 8: Data Collection Final Report*. Substance Abuse and Mental Health Services Administration; 2023.

28. Centers for Disease Control and Prevention. *Behavioral Risk Factor Surveillance System: 2020 Summary Data Quality Report.* 2021. Accessed February 27, 2024. https://www.cdc.gov/brfss/annual_data/2020/pdf/2020-sdqr-508.pdf

29. Centers for Disease Control and Prevention. *Behavioral Risk Factor Surveillance System: 2022 Summary Data Quality Report*. 2023. Accessed February 27, 2024. https://www.cdc.gov/brfss/annual_data/2022/pdf/2022-DQR-508.pdf

© 2024 Wright E et al. *JAMA Network Open*.
