# Supplementary material for: Prevalence of and Inequities in Poor Mental Health Across 3 US Surveys, 2011 to 2022
Source: JAMA Netw Open. 2025 Jan 15;8(1):e2454718. doi: 10.1001/jamanetworkopen.2024.54718 (PMC11736504; doi:10.1001/jamanetworkopen.2024.54718)
Supplement: Supplement 2. — Data Sharing Statement [file jamanetwopen-e2454718-s002.pdf]

Data Sharing Statement

Wright. Prevalence of and Inequities in Poor Mental Health Across 3 US Surveys, 2011 to 2022. *JAMA Netw Open*. Published January 15, 2025.
doi:10.1001/jamanetworkopen.2024.54718

Data
Data available: Yes
Data types: Deidentified participant data, Other (please specify)
Additional Information: All data analyzed for this study are publicly available from the Behavioral Risk Factor Surveillance System (https://www.cdc.gov/brfss/annual_data/annual_data.htm), the National Survey on Drug Use and Health (https://www.datafiles.samhsa.gov/dataset/national-survey-drug-use-and-health-2022-nsduh-2022-ds0001; use drop-down list to choose data from another year), and—for the National Health Interview Survey—from IPUMS Health Surveys (https://nhis.ipums.org/nhis/).
How to access data: All data analyzed for this study are publicly available from the Behavioral Risk Factor Surveillance System (https://www.cdc.gov/brfss/annual_data/annual_data.htm), the National Survey on Drug Use and Health (https://www.datafiles.samhsa.gov/dataset/national-survey-drug-use-and-health-2022-nsduh-2022-ds0001; use drop-down list to choose data from another year), and—for the National Health Interview Survey—from IPUMS Health Surveys (https://nhis.ipums.org/nhis/).
When available: With publication

Supporting Documents
Document types: None

Additional Information
Who can access the data: All data analyzed for this study are publicly available.
Types of analyses: For any purpose.
Mechanisms of data availability: All data analyzed for this study are publicly available, without investigator support.
